# Interventions for treating patients with chikungunya virus infection-related rheumatic and musculoskeletal disorders: A systematic review

**DOI:** 10.1371/journal.pone.0179028

**Published:** 2017-06-13

**Authors:** Arturo Martí-Carvajal, Pilar Ramon-Pardo, Emilie Javelle, Fabrice Simon, Sylvain Aldighieri, Hacsi Horvath, Julia Rodriguez-Abreu, Ludovic Reveiz

**Affiliations:** 1 Department of Public Health, Universidad de Carabobo, Valencia, Venezuela; 2 Communicable Diseases and Health Analysis (CHA), Pan American Health Organization, Washington DC, United States of America; 3 Hôpital d’Instruction des Armées Alphonse Laveran, Marseille, France; 4 PAHO Health Emergencies Department (PHE), Pan American Health Organization, Washington DC, United States of America; 5 Department of Epidemiology and Biostatistics, University of California, San Francisco, California, United States of America; 6 Knowledge Management, Bioethics and Research (KBR), Pan American Health Organization, Washington DC, United States of America; SERGAS and IDIS, SPAIN

## Abstract

**Background:**

Chikungunya virus infection (CHIKV) is caused by a mosquito-borne alphavirus. CHIKV causes high fever and painful rheumatic disorders that may persist for years. Because little is known about interventions for treating CHIKV-related illness, we conducted a systematic review.

**Methods:**

We used Cochrane methods. We searched PubMed, EMBASE, Cochrane Library, LILACS and other sources from the earliest records to March 2016. We had no language restrictions. We included randomized controlled trials assessing any intervention for treating acute or chronic CHIKV-related illness. Our primary outcomes were pain relief, global health status (GHS) or health related quality of life (HRQL), and serious adverse events (SAEs). We assessed bias risk with the Cochrane tool and used GRADE to assess evidence quality.

**Results:**

We screened 2,229 records and found five small trials with a total of 402 participants. Patients receiving chloroquine (CHQ) had better chronic pain relief than those receiving placebo (relative risk [RR] 2.67, 95% confidence interval [CI] 1.23 to 5.77, N = 54), but acute pain relief was marginally not different between groups (mean difference [MD] 1.46, 95% CI 0.00 to 2.92, N = 54). SAEs were similar (RR = 15.00, 95% CI 0.90 to 250.24, N = 54). Comparing CHQ with paracetamol (PCM), CHQ patients had better pain relief (RR = 1.52, 95% CI 1.20 to 1.93, N = 86). Compared with hydroxychloroquine (HCHQ), disease-modifying anti-rheumatic drugs (DMARDs) reduced pain (MD = -14.80, 95% CI -19.12 to -10.48, N = 72). DMARDs patients had less disability (MD = -0.74, 95% CI -0.92 to -0.56, N = 72) and less disease activity (MD = -1.35; 95% CI -1.70 to -1.00; N = 72). SAEs were similar between DMARDs and HCHQ groups (RR = 2.84, 95% CI 0.12 to 67.53, N = 72). Comparing meloxicam (MXM) with CHQ, there was no difference in pain relief (MD = 0.24, 95% CI = -0.81 to 1.29; p = 0.65, N = 70), GHS or HRQL (MD = -0.31, 95% CI -2.06 to 1.44, N = 70) or SAEs (RR = 0.85, 95% CI 0.30 to 2.42, N = 70). Finally, a four-arm trial (N = 120) compared aceclofenac (ACF) monotherapy to ACF+HCHQ, ACF+ prednisolone (PRD), or ACF+HCHQ+PRD. Investigators found reduced pain (p<0.001) and better HRQL (p<0.001) in the two patient groups receiving PRD, compared to those receiving ACF monotherapy or ACF+HCHQ. Trials were at high risk of bias. GRADE evidence quality for all outcomes was very low.

**Conclusion:**

Results from these small trials provide insufficient evidence to draw conclusions about the efficacy or safety of CHIKV interventions. Physicians should be cautious in prescribing and policy-makers should be cautious in recommending any intervention reviewed here. Rigorous trials with sufficient statistical power are urgently needed, with results stratified by disease stage and symptomology.

## Introduction

Chikungunya virus infection (CHIKV) is caused by an alphavirus transmitted by *Aedes aegypti* and *Aedes albopictus* mosquitoes [1]. Three reports from the mid-1950s describe an outbreak of viral disease observed in the Makonde Plateau of Tanganyika (now in southern Tanzania) in 1952–1953 [2], the environmental and epidemiologic aspects of that outbreak [3] and the virology and other characteristics of the pathogen itself [4]. Acute, severe joint pain was the most prominent clinical feature of the disease [2]. Given the distinctive severity and sudden onset of this pain, the Kimakonde people primarily affected by the 1952–1953 outbreak called it *chikungunya*, “that which bends up” [2]. CHIKV’s chronic manifestations and sequelae were not described until more than two decades later [5–7].

Until 2005, CHIKV circulated only in intertropical Africa (two genotypes) and in Asia (one genotype). Although known CHIKV outbreaks in Africa were relatively small, there were several very large CHIKV outbreaks in Asia, notably in India and Thailand [8]. Nearly one-third of the population of Bangkok, Thailand was infected with CHIKV in 1962 [8]. Even so, CHIKV control was not generally considered to be a high priority in public health and global health efforts.

This perspective changed with the 2005–2006 CHIKV outbreak in the southwest Indian Ocean region, specifically in the Comoros islands, Mauritius, and the French overseas territories of La Réunion and Mayotte. During this outbreak a new epidemiologic profile for CHIKV was described, with high attack rates [9–11]. More than one-third of Réunion’s population experienced CHIKV-related symptoms and CHIKV was suspected in the deaths of more than 200 persons there [11]. CHIKV antibodies were found in sera of nearly two-thirds of the population on the island of Grande Comore in Comoros [10]. The epidemic continued to spread to other settings. By April 2016, over two million suspected or confirmed cases of CHIKV had been reported since 2005 in 60 countries of Asia, Africa, Europe and the Americas [12], though the true number of cases could be much higher. More than half of reported cases were seen in 44 countries and territories of the Caribbean Islands, Central America, South America and North America [13], with attack rates in some areas exceeding 60% [14].

More than half of CHIKV-infected patients typically experience symptoms [9], with estimates ranging up to 97% of patients [8]. Symptoms emerge suddenly, within 2–12 days of the vector’s bite [8]. CHIKV’s symptomology and multifaceted clinical course have been described in detail [15], and are summarized below.

After the initial incubation period, patients may experience the following three stages:

1. an acute viremic stage: typically characterized by the triad of fever, severe polyarthritis and rash, generally resolving by the end of the third week;
2. a post-acute stage: with continued severe arthritis and with the addition of periarticular and synovial inflammation, peripheral vascular disorders, neuropathy, neuropsychiatric disorders or other clinical manifestations; generally persisting to around the end of the third month; and
3. a chronic stage: when the range of rheumatic, musculoskeletal and other symptoms observed in the post-acute stage persists beyond three months; it has been reported persisting in patients up to 15 years after acute CHIKV [16].

The proportion of CHIKV patients progressing to the chronic stage has differed among outbreak settings. While only 4.1% of patients in India reported persistent rheumatic pain 12 months after CHIKV onset [17], far more patients have progressed to the chronic stage elsewhere. For example, the proportion of those reporting joint disorders at 15–18 months was much higher (57%-64%) in Réunion [18,19] as was the proportion (78.6%) reporting persistent musculoskeletal symptoms in Mauritius at 27.5 months [20]. Along with older age and CHIKV antibody titres, severity of illness in the acute stage may predict progression to the chronic stage [21]. While a minority of CHIKV patients may not progress to the chronic stage, even the acute and post-acute stages result in very significant physical pain and disability, psychological suffering, worsened quality of life and reduced wellbeing for a period of three months. In patients progressing to the chronic stage, this pain, suffering and disability may continue for many years [20,22].

Little is known about appropriate treatments for CHKV-related acute and chronic illness. At the time of the 2005–2006 southwest Indian Ocean region epidemic there was only one published report examining any approach to treating CHIKV-related illness; a non-randomized pilot study in 10 patients [16]. Clinicians treating CHIKV patients thus had no evidence-based treatment plans available to them. They had to rely on standard and adjuvant approaches used by family doctors, rheumatologists and pain specialists in treating other diseases that manifest with similar symptoms. In the dozen years since that time, clinicians worldwide have accrued a great deal of experience in treating CHIKV patients. Given the possibility that some of this work has been tested in randomized controlled trials (RCTs), our objective was to conduct a systematic review of the efficacy and safety of interventions for treating CHIKV-related acute and chronic illness.

## Methods

We followed Cochrane Collaboration review methods [23] and report our review according to the Preferred Reporting Items for Systematic Reviews and Meta-Analyses (PRISMA) guidance [24] available in the S1 File. The protocol of this review was registered in PROSPERO [25], number CRD42015019397.

### Search methods

We comprehensively searched the scientific literature to identify studies assessing the effects of therapeutic interventions for treating acute and persisting CHIKV-related rheumatic illness. Using a range of relevant keywords, database-specific syntax and validated search filters, we conducted structured searches in the following sources from earliest records up to March 2016: PubMed, EMBASE, the Cochrane Library, and LILACS. Using PubMed’s indexing syntax, search terms included the following:

“Chikungunya Fever”[MeSH] OR “chikungunya virus”[MeSH] OR chikungunya[tw] OR CHIKV[tw] OR Aedes aegypti[tw] OR Aedes albopictus[tw] OR Ae aegypti[tw] OR Ae albopictus[tw]

We adapted our search strategy to the requirements of each database and combined this algorithm with validated filters to retrieve clinical trials.

We also searched WHO’s International Clinical Trials Registry Platform (ICTRP) to identify past and ongoing trials indexed with the keyword “chikungunya.” We examined bibliographic references of both included and excluded studies in an effort to find further relevant papers. We searched for unpublished studies in Google Scholar, ISI Conference Proceedings, OPENSIGLE, and databases of dissertations and theses. We reached out to authors and relevant key stakeholders to identify unpublished randomized controlled trials and related additional data from manuscripts. S2 File provides additional detail.

### Inclusion and exclusion criteria

We included randomized controlled trials assessing the clinical benefits and harms of the interventions for treating acute or persisting rheumatic and musculoskeletal disorders in patients infected with CHIKV. We had no restrictions in terms of publication language or duration of study follow-up. We excluded non-randomized trials and observational studies.

#### Population

Eligible trials randomized symptomatic participants of any age, infected with probable or confirmed CHIKV, to intervention or control conditions. Participant illness could be at any stage of the CHIKV clinical course (i.e. acute, post-acute or chronic).

#### Interventions

Eligible trials compared any of the following broad types of interventions to control conditions and reported quantitative results.

- Analgesics: e.g. paracetamol (PCM)
- Antiviral therapy
- Chloroquine (CHQ)
- Corticosteroids: e.g. prednisolone (PRD)
- Disease-modifying anti-rheumatic drugs (DMARDs): e.g. methotrexate (MTX), sulfasalazine (SSZ), hydroxychloroquine (HCHQ)
- Homeopathy
- Immunomodulation biologic agents
- Leflunomide
- Non-steroidal anti-inflammatory drugs (NSAIDs): e.g. aceclofenac (ACF), meloxicam (MXM)
- Opioid drugs
- Physiotherapy
- Vitamin D

#### Comparators

Eligible trials could assess any pharmacological intervention independently of its administration route, dosage or duration of treatment, compared to any control group condition. Control conditions could be any interventional, placebo or sham treatment used by study investigators, or no treatment.

#### Outcomes

Eligible trials reported data for any of the following primary outcomes:

1. Pain relief. Studies could report the rate of patients experiencing pain relief or studies measuring this outcome through any validated scale or questionnaire (e.g. persistence of arthralgia at follow-up).
2. Global health status (GHS) or health-related quality of life (HRQL), measured with validated scales or questionnaires.
3. Serious adverse events (SAEs). We assessed rates of SAEs arising from intervention or control condition drugs. In defining SAEs, we accepted the definition of the International Conference on Harmonisation (ICH) Guidelines for Good Clinical Practice [26]. We also considered any symptom or sign related to gastric mucosal damage as an SAE.

In trials reporting data for any of the above primary outcomes, we also collected available data on the following secondary outcomes:

1. Number of days loss of work or school
2. Tender joint count
3. Swollen joint count
4. Physician’s global assessment
5. Patient’s global assessment

### Study selection and data extraction

After excluding duplicate records, two reviewers (AMC and LR) independently examined the titles, abstracts and keywords of all studies retrieved in the searches. We obtained full text articles of any records for which eligibility was unclear as well as those that clearly seemed to meet inclusion criteria. After reviewing these full text articles, we made final decisions about study eligibility for inclusion in the review. We planned for a neutral third reviewer (JR) to adjudicate any disagreement.

Using a pre-designed data extraction form, two reviewers (AMC and LR) independently extracted the following types of relevant data from included trials:

- Study details: Dates when research was conducted, geographic location, participant inclusion criteria, funding sources, publication date
- Participant characteristics: Age, gender, ethnicity, socioeconomic status
- CHIKV details: Diagnostic test used to confirm CHIKV, definitions of CHIKV chronicity
- Intervention details: Type, duration, method used to measure
- Outcome details: Type of outcome, outcome assessment method, type of statistical analysis, adjustment variables
- Bias assessment details: Data necessary to assess the risk of bias, as described below

In the event of inconsistencies or disagreements in the extracted data, we planned for a neutral third reviewer (JR) to adjudicate.

### Risk of bias assessment

We assessed the risk of bias for each trial with the Cochrane Collaboration instrument, which evaluates following sources of bias [23]:

- Selection bias (how the random sequence was generated, and allocation concealment);
- Performance bias (blinding of participants and/or personnel);
- Detection bias (blinding of outcome assessments);
- Attrition bias (incomplete outcome data);
- Reporting bias (assessment of selective reporting of outcomes)

Where bias risk was unclear, we contacted study authors to obtain additional information.

### Data synthesis and analysis

#### Effect measures

For continuous outcomes (e.g. change in patient-reported pain relief or quality of life), we estimated mean differences (MD) with their 95% confidence intervals (CI). For binary outcomes (e.g. the rate of patients experiencing SAEs, we estimated relative risks (RR) with their 95% CIs.

#### Missing data

Our analysis was by the intention-to-treat principle. Where trials reported outcomes in an as-treated or per-protocol analysis, we counted dropouts and losses to follow-up as failed treatment.

#### Meta-analysis

Had we pooled data in meta-analysis, we planned to use the I^2^ statistic to assess statistical heterogeneity. We considered significant heterogeneity to exist if I^2^ were >50% [27]. We planned to use a random effects meta-analytic model for such analyses. In pooled data with I^2^ ≤50%, we would have used a fixed-effects model. If any meta-analysis included data from ≥10 trials, we would have used a funnel plot to assess the risk of publication bias. Finally, we planned to conduct sensitivity analyses for the risk of attrition bias (at the level of ≥15% withdrawal rate). Our intention was to use Cochrane’s RevMan software [28] for all statistical analyses.

#### Subgroup analysis

Had data permitted, we planned to conduct the following subgroup analyses for primary outcomes:

1. Younger patients (age <18 years) versus older patients (age ≥55 years).
2. Probable CHIKV patients (without biological confirmation) versus confirmed CHIKV patients.
3. Type of interventions.
4. Trials with low risk of bias versus trials with high risk of bias. High risk of bias was defined as unclear risk for random sequence generation, unclear risk for allocation concealment and unclear or high risk of bias for blinding of participants, personnel, and outcome assessors.

#### Quality of the evidence

We used GRADE methods to assess evidence quality for each primary outcome [29]. “Quality of evidence” is defined as “the extent of our confidence that the estimates of effect are correct" [23]. There are four levels for evidence quality: high, moderate, low, and very low. Randomized trial data are at first considered to be of high quality but can be graded down for high risk of bias, indirectness of evidence, inconsistency, imprecision and publication bias. Observational study data start out as low quality evidence, but can potentially be graded up if there is a large effect, a dose-response gradient or if plausible confounding would increase confidence in an effect estimate (e.g. where patients who were more sick at baseline experienced better outcomes than healthier comparator patients). We used the GRADEpro software [30] to grade evidence quality.

## Results

### Results of searches

**Fig 1:**
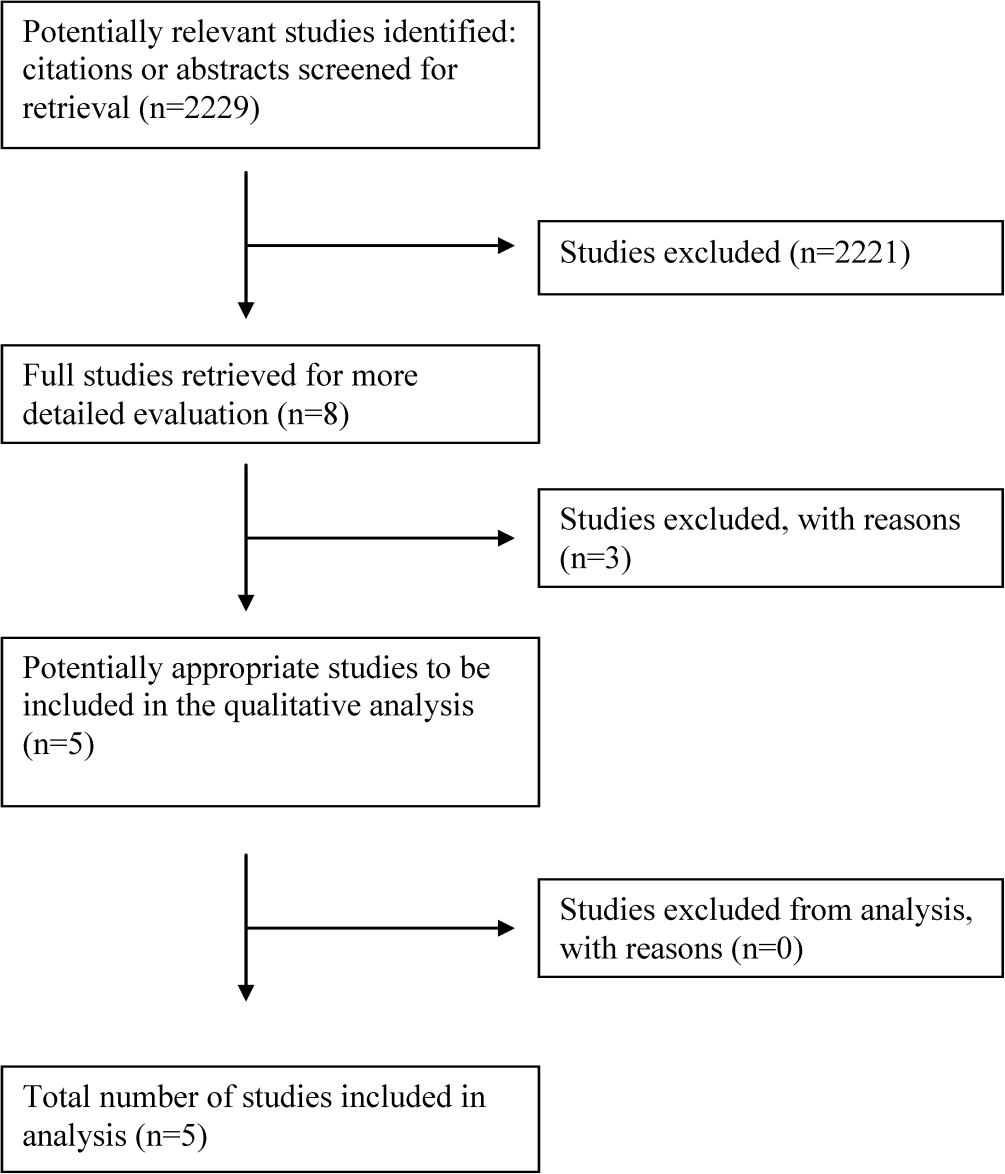
Flowchart diagram for the selection of studies.

Our searches brought 2,229 unique references, after removing duplicate records. We applied the inclusion criteria to the titles, abstracts and keywords of all records and excluded 2,221 records. We examined the full text articles for eight records. Five trials [31–35] with 402 total participants met our inclusion criteria. The other three studies were excluded because they were not randomized trials [36] or they repeated data that was reported more completely in one of our included trials [37–38]. Fig 1 shows our screening and study selection process.

### Study characteristics

Included trials were conducted in Réunion [31] and in India [32–35]. All but one trial used serological tests to diagnose CHIKV; one Indian trial [32] based diagnoses on clinical and epidemiologic characteristics.

All trials were conducted in adults (age ≥18 years). One trial [31] tested the effectiveness of treatment in the acute phase of CHIKV infection and assessed persisting symptoms. The remaining trials [32–35] tested interventions in patients with musculoskeletal pain and arthritis persisting after the acute and post-acute stages.

Three trials [31–32, 35] tested the effectiveness of CHQ in reducing symptoms, but each trial’s comparator was different. One trial [31] used placebo; another trial [32] used PCM. A third trial [33] tested the effectiveness of MXM in reducing symptoms, with the effect of CHQ serving as comparator. None of these trials reported the type of CHQ used. One four-arm trial [34] compared ACF monotherapy versus ACF plus HCHQ, ACF plus PRD or ACF plus HCHQ plus PRD. tested the effectiveness of DMARDs in combination (MTX, SSZ and HCHQ) for relieving CHIKV-related rheumatic symptoms, compared to the effect of HCHQ monotherapy. Finally, one trial [35] tested the effectiveness of DMARDs in combination (MTX, SSZ and HCHQ) for relieving CHIKV-related rheumatic symptoms, compared to the effect of HCHQ monotherapy. Table 1 shows summary characteristics of each included trial. S1 Table provides much additional detail.

**Table 1:** Summary characteristics of included trials.

Study	Setting	Participants	Timing	Intervention	Comparator	Primary outcomes assessed
Ahmed 2012 [32]	India	N = 86	Post-acute stage	CHQ	PCM	Pain relief (assessed with VAS)
Chopra 2004 [33]	India	N = 70	>6 weeks after CHIKV symptom onset	MXM	CHQ	Pain severity (assessed with VAS)
De Lamballerie 2008 [31]	Réunion	N = 54	<48 hours of CHIKV symptom onset	CHQ	Placebo	Arthralgia at day 200; SAEs
Padmakumar 2009 [34]	India	N = 120	<6 weeks of CHIKV fever onset	a. ACF	b. ACF+HCHQ c. ACF+PRD d. ACF+HCHQ+PRD	Pain severity and HRQL (both assessed with VAS)
Ravindran 2011 [35]	India	N = 72	>1 year after CHIKV fever onset	DMARDs	HCHQ	Pain relief, disability, disease activity

Legend: VAS: Visual analog scale. CHQ: chloroquine, PCM: paracetamol, MXM: meloxicam, ACF: aceclofenac, HHQ: hydroxychloroquine, PRD: prednisolone, DMARDs: disease-modifying anti-rheumatic drugs.

### Risk of bias

All included trials were at high risk of bias (due to flaws in design and execution) or unclear risk of bias (due to poor reporting). Four trials did not clearly report methods used for randomization. No trial reported clearly whether allocation to treatment group was concealed. Three trials were at high risk of bias for lack of blinding in patients, study personnel and outcome assessors. Two trials were at high risk of bias for selective outcome reporting and three trials were at high risk of other types of bias. Table 2 shows bias risk for each domain in included trials. S2 Table provides additional detail.

**Table 2:** Summary risk of bias assessment in included trials.

Study	Sequence generation	Allocation concealed	Blinding of participants and personnel	Blinding of outcome assessment	Incomplete outcome data	Selective reporting	Other bias
Ahmed 2012 [32]	Low risk	Unclear risk	High risk	High risk	Low risk	High risk	Bias in presenting data
Chopra 2004 [33]	Unclear risk	Unclear risk	Unclear risk	Unclear risk	Low risk	Low risk	High risk
De Lamballerie 2008 [31]	Unclear risk	Unclear risk	Unclear risk	Unclear risk	Unclear risk	Unclear risk	Unclear risk
Padmakumar 2009 [34]	Unclear risk	Unclear risk	High risk	High risk	Low	High risk	High risk
Ravindran 2011 [35]	Unclear risk	Unclear risk	High risk	High risk	Unclear risk	Unclear risk	Low

### Intervention effects

#### CHQ versus placebo (one trial)

De Lamballerie and colleagues [31] randomized 54 participants with biologically-proven acute stage CHIKV (<48 hours of symptom onset) to CHQ or placebo. Investigators interviewed most participants by telephone at 200 days follow-up. They reported 16 (61%) CHQ participants with arthralgia compared to six (23%) 23% in the placebo group (p<0.01). Patients receiving CHQ had better chronic pain relief than those receiving placebo (RR 2.67, 95% CI 1.23 to 5.77), but acute pain relief was marginally not different between groups (MD 1.46, 95% CI 0.00 to 2.92). SAEs were similar between groups (RR = 15.00, 95% CI 0.90 to 250.24).

Patients reported 4.3 days mean duration of febrile arthralgia in the CHQ group versus 3.9 days in the placebo group. Investigators declared there was no significant difference between groups and did not show a p-value, standard deviation or standard error.

#### CHQ versus PCM (one trial)

Ahmed and colleagues [32] randomized 86 participants in the post-acute stage of CHIKV to CHQ or PCM. Participants were followed for eight days. CHQ group patients had better pain relief than those receiving PCM (RR = 1.52, 95% CI 1.20 to 1.93).

#### MXM versus CHQ (one trial)

Chopra and colleagues [33] randomized 70 participants to MXM or CHQ, at least six weeks after onset of CHIKV symptoms. Participants were followed for 24 weeks. Results were inconclusive in regard to pain relief (MD = 0.24, 95% CI = -0.81 to 1.29), GHS or HRQL (MD = -0.31, 95% CI -2.06 to 1.44) or SAEs (RR = 0.85, 95% CI 0.30 to 2.42). For the secondary outcome of tender joint count, results were again inconclusive (MD = -0.45, 95% CI = -7.30 to 6.40), as was also the case with swollen joint count (MD = 0.57, 95% CI = -0.33 to 1.47).

#### ACF monotherapy versus ACF plus other drugs (one trial)

Padmakumar and colleagues [34] ran a trial in which 120 patients were randomized within six weeks of CHIKV fever onset to one of four regimens: 1) ACF monotherapy; 2) ACF plus HCHQ; 3) ACF plus PRD; or 4) ACF plus HCHQ plus PRD. Participants were followed for 12 weeks. Investigators found reduced pain (p<0.001) and better HRQL (p<0.001) in either of the two patient groups receiving PRD, compared to those receiving ACF monotherapy or ACF plus HCHQ.

#### DMARDs in combination versus HCHQ monotherapy (one trial)

Ravindran and colleagues [35] randomized 72 participants to DMARDs in combination or to HCHQ monotherapy. Patients were randomized at least one year after initial onset of CHIKV fever and were assessed every four weeks for 24 weeks. Compared with patients receiving HCHQ, DMARD patients had reduced pain (MD = -14.80, 95% CI -19.12 to -10.48), less disability (MD = -0.74, 95% CI -0.92 to -0.56) and less disease activity (MD = -1.35; 95% CI -1.70 to -1.00). SAEs were similar between the DMARD and HCHQ groups (RR = 2.84, 95% CI 0.12 to 67.53).

Table 3 summarizes results for all reported primary outcomes in the five trials. S1 Table provides additional detail.

**Table 3:** Quantitative results for reported primary outcomes.

Study	Intervention	Outcome	Effect (95% CI)
Ahmed 2012 [32]	CHQ versus PCM	Pain relief	RR = 1.52 (1.20 to 1.93)
Chopra 2004 [33]	MXM versus CHQ	Pain relief	MD = 0.24 (-0.81 to 1.29)
“	“	GHS or HRQL	MD = -0.31 (-2.06 to 1.44)
“	“	SAEs	RR = 0.85 (0.30 to 2.42)
De Lamballerie 2008 [31]	CHQ versus placebo	Arthralgia	p<0.01*
“	“	Chronic pain relief	RR 2.67 (1.23 to 5.77)
“	“	Acute pain relief	MD-1.46 (0.00 to 2.92)
“	“	SAEs	RR = 15.00 (0.90 to 250.24)
Padmakumar 2009 [34]	ACF regimens containing PRD versus ACF regimens without PRD*	Pain relief	p<0.001*
“	“	HRQL	p<0.001*
Ravindran 2011 [35]	DMARDs	Pain	MD = -14.80 (-19.12 to -10.48)
“	“	Disability	MD = -0.74 (-0.92 to -0.56)
“	“	Disease activity	MD = -1.35 (-1.70 to -1.00)
“	“	SAEs	RR = 2.84 (0.12 to 67.53)

*As reported by investigators.

### Quality of the evidence

Evidence quality for all outcomes was very low. According to the GRADE system for this rating suggests that we can have very little confidence in these estimates of effect. The true effects of these interventions on all outcomes are likely to be substantially different [29]. We have already described the high risk of bias seen in all trials across several bias domains, but there were additional reasons to grade down evidence quality for all reported outcomes. Among these are the small sample sizes in all trials, which results in statistical imprecision and wide CIs. This imprecision may be compounded when outcome event rates are low (e.g. as with SAEs). See our complete GRADE analyses (with annotations for our judgments) in S3 Table.

## Discussion

Despite comprehensive searches in a range of databases, no restrictions on language and rigorous review methods, we found only five small trials exploring several interventions to reduce pain and improve other outcomes in patients suffering from acute or chronic CHIKV-related illness. Evidence from these trials is unreliable and we can have only minimal confidence that reported effect estimates are somewhat close to the true effects. Although CHIKV-related illness has caused much long-lasting and persistent suffering in some millions of patients worldwide in recent years, it is not usually a fatal syndrome, with most mortality seen in elderly or immunocompromised subpopulations. Perhaps this helps to explain why the scientific community has not responded to CHIKV with large and rigorous randomized interventional studies. Funding for health research is always limited, of course, and this situation is common to other such neglected tropical diseases. Few resources are ever committed to such conditions unless they begin to make an epidemiologic impact on morbidity and mortality in high income countries, as was the case with HIV. However, the world needs to take decisive action on CHIKV. The rapid emergence of CHIKV in the Americas and its progress throughout the region is very worrisome.

This systematic review on interventions for treating patients with either acute or post-acute or chronic rheumatic disorders after a CHIKV infection identified five RCTs involving 402 participants.

The main results of this systematic review are:

1. The clinical type of rheumatic disorders (synovitis, tendonitis, tenosynovitis, other) are unknown in most RCT.
2. In participants with biologically-proven CHIK acute infection it is unknown the clinical effectiveness of chloroquine compared with placebo.
3. Chloroquine (salt type reported no by any trial) compared with paracetamol seems to be effective to pain relief, however this result has a very low quality of evidence. Furthermore we identified a major limitation of this study because the paracetamol doses are suboptimal (500 mg/24h instead of 500–1000 mg/ 6-8h).
4. DMARDs combination compared with hydroxychloroquine alone also seems to be effective to pain relief and to improve the quality of life.
5. Safety profile is unknown.
6. We were not able to perform a meta-analysis due to different comparisons and other issues.
7. Some evidence emerges from small trials with high risk of bias; thus, quality of evidence from all trials was rated as very low (S3 Table. GRADE evidence profiles).

### Overall completeness and applicability of evidence

Despite the high number of papers retrieved in the systematic search, only five were RCT aiming to assess the impact on the long-term post-CHIK rheumatic and general outcome. The initial underestimation, the lack of clinical classification of the persisting post-CHIK rheumatic disorders at the time of the studies’ start probably conditioned the clinical design for testing of therapeutic interventions. Most of the knowledge on the CHIK infection has been developed after 2005, specifically on the long-term symptoms or sequelae. This revision clearly highlights the need of RCT to assess effective interventions for the persisting rheumatic disorders after CHIKV infection.

The lack of standard case definition for persisting rheumatic manifestations, and the non-identification of a subgroup of patients with well-defined chronic inflammatory rheumatism, impedes the comparison of results among different studies. Heterogeneity of chronic manifestations in patients should be dismembered using clinical, biological and imaging criteria and according recent classifications [13] before testing therapeutic interventions.

It was not possible to perform the subgroup analysis for younger patients versus older patients, since the cases in the assessed studies were over 18 years old. The frequency of chronic rheumatic manifestations in young patients seems to be anecdotal.

This systematic review has found inconclusive evidence on all interventions reported into included trials. It is mostly explained by heterogeneity of assessed experimental interventions, and insufficient data supplied by trial authors. Heterogeneity on definitions and measurement outcomes, and different interventions hindered any chance of pooling data. For example, Chopra et al [33] reported pain relief with mean difference whilst Ahmed et al. reported this outcome using a dichotomous approach [32], and De Lamballerie measured that outcome with both approaches [31]. The transformation of a continuous outcome (response) to a binary outcome may lead to dichotomization bias which increases the risk of either false or negative error and generates bias [39].

### Limitations of this review

The main limitation of this review is due to the very smallness of sample size, which is associated with either overestimates of effect size or low reliability of results [40]. The low sample size is connected with generation of excess significance, winner’s course, and vibration of effects [41].Therefore, due to the paucity of evidence; we can’t recommend anything; it’s all uncertain.

## Authors' conclusions

### Implications for practice

This systematic review for assessing the clinical benefits and harms of interventions for preventing or treating persisting rheumatic disorders in patients with an infection caused by the CHIKV has identified inconclusive evidence either to support or reject any intervention for treating these patients. This conclusion emerges from five small trials with high risk of bias. Therefore, physicians should be cautious when prescribing to treat patients with chronic rheumatic disorders caused by CHIKV. Rheumatic assessment and progressive scaling up of the therapy (from analgesics, NSAIDs to corticosteroids) should be strictly followed before introducing advanced line treatments as DMARDs. These findings are aligned with recent guidelines based on experts’ consensus [15].

### Implications for research

There is a need of powered randomized clinical trials with high quality methodology to assess clinical benefits and harms of interventions for preventing or treating persisting rheumatic disorders in patients with an infection caused by the CHIKV. The potential trial should be planned according to SPIRIT recommendations [42–44] and reported according to CONSORT statement [45]. The trial should include patient-centered outcomes such as it is recommended by The Patient-Centered Outcomes Research Institute (PCORI) [46] Outcome Measures in Rheumatology (OMERACT) [47] and by the International Conference on Harmonisation.

## Supporting information

S1 File PRISMA 2009 checklist. (DOC)

S2 File PubMed search strategy. (DOCX)

S1 Table Characteristics of included studies. (DOCX)

S2 Table Risk of bias assessment. (DOCX)

S3 Table Summary of findings tables. GRADE evidence profile. (DOCX)
