# Supplementary material for: Interventions for treating patients with chikungunya virus infection-related rheumatic and musculoskeletal disorders: A systematic review
Source: PLoS One. 2017 Jun 13;12(6):e0179028. doi: 10.1371/journal.pone.0179028 (PMC5469465; doi:10.1371/journal.pone.0179028)
Supplement: S2 File — (DOCX) [file pone.0179028.s002.docx]

**Pubmed Search Strategy**

(chikungunya Fever [mh] OR chikungunya virus [mh] OR chikungunya [tiab] OR CHIKV [tiab] OR post-chikungunya [tiab]) AND (randomized controlled trial [pt] OR controlled clinical trial [pt] OR randomized [tiab] OR placebo [tiab] OR drug therapy [sh OR randomly [tiab] OR trial [tiab] OR groups [tiab])
