# Supplementary material for: Interventions for treating patients with chikungunya virus infection-related rheumatic and musculoskeletal disorders: A systematic review
Source: PLoS One. 2017 Jun 13;12(6):e0179028. doi: 10.1371/journal.pone.0179028 (PMC5469465; doi:10.1371/journal.pone.0179028)
Supplement: S1 Table — (DOCX) [file pone.0179028.s003.docx]

**Table 1. Characteristics of included studies**

| **Reference** | **Methods** | **Participants** | **Interventions** | **Outcomes** | **Notes** |
| --- | --- | --- | --- | --- | --- |
| Chopra 2014^(44)^  Country: India | Parallel design  Two arms | Age (mean, standard deviation or standard error mean: not reported).  Chloroquine: 50.2  Meloxicam: 45.4  Gender (Female)  Chloroquine: 63.15%  Meloxicam: 97%.  Type of population: very poor farming community.  Enrolled: 509  Randomized: 70  Time after disease onset: more than 6 weeks’ duration following the onset of CHIKV infection.  Stage: early persistent musculoskeletal pain and arthritis following CHIK virus infection.  **Inclusion criteria:** a) persistent musculoskeletal pain and arthritis of more than 6 weeks’ duration following the onset of CHIKV infection b) seropositivity for IgG and/or IgM anti-CHIK antibody, c) a maximum visual analog scale (VAS) score for pain of 4 cm (0–10-cm scale).  Exclusion criteria: history of rheumatoid arthritis (RA) or any inflammatory arthritis disorder, drug hypersensitivity, recent severe illness (other than  CHIKV infection), unwillingness to participate, and any other condition (based on the investigator’s discretion). | Chloroquine (250 mg tablet) once daily after lunch.  Meloxicam (7.5 mg tablet) once daily after lunch  Co-intervention: oral acetaminophen (500-mg tablet) as-needed basis patient.  Aim: antiviral-inflammatory. | Primary outcome: maximum pain severity in the musculoskeletal tissues and joints during the preceding 24 hours, recorded on a 100-cm  VAS.  Secondary outcomes:  a) tender  joint count in 68 diarthrodial joints;  b) a swollen joint count in 66  joints (excluding hips);  c) physician’s global assessment of disease  activity and patient’s global assessment of disease activity  d) functional assessment  e) Oral acetaminophen intake  f) Adverse events  g) Routine laboratory assessments  h) Cytokine assays  Follow-up: 24 weeks | Identifier trial number: Clinical Trial Registry India no.: 2010/091/000208  Sample size estimation a priori: No.  Conduction trial date: not stated  Sponsor: Arthritis Research Care Foundation Center for Rheumatic Diseases, India, and the Indian Council of Medical Research, Government of India (grant CHIKV-ICMR PROJECT05/8/7/20/2006-ECD-I).  Role of sponsor: not stated.  Conflict of interest: not stated. |
| De Lamballerie 2008 ^(41)^  Country: French Reunion Island (Indian Ocean) | Parallel design  Two arms | 54 adult patients (18–65 years old, men and women) with a biologically confirmed diagnosis of acute CHIK infection (viremia quantified by real-time reverse-transcriptase polymerase chain reaction, and seroconversion between day 1 and day 16 of the protocol).  Stage: acute  Time after disease onset: acute febrile arthralgia, and diagnosed within less than 48 hours.  Inclusion criteria: a) having a body weight equal to or higher than 60 kg b) a typical presentation of acute CHIK disease (defined by acute febrile arthralgia), and diagnosed within less than 48 hours. Exclusion criteria included pregnancy, classical counterindications to chloroquine, renal insufficiency,  retinopathy, and celiac disease | Chloroquine: 27 patients.  Dose: 600 mg at day 1, 600 mg at days 2 and 3, and 300 mg at days 4 and 5 (total dose: 2,400 mg; duration of treatment:  5 days during the acute phase).  Placebo: 27 patients.    Aim: antiviral-inflammatory and immunomodulation. | Presence of arthralgia at day 200.  Acute adverse events. | The main criterion studied to evaluate the efficacy of the treatment was the duration of febrile arthralgia. The decrease of viremia between day 1 and day 3 was quantified.  Identifier trial number: not stated.  Identifier trial number: not stated.  Sample size estimation a priori: No.  Conduction trial date: August 2012  Sponsor: not stated  Role of sponsor: not applied.  Conflict of interest: not declared. |
| Ahmed 2012 ^(45)^  Country: India. | Parallel design  Two arms | Sample size: 86  Age (years – median, interquartile range):  Chloroquine: 40.2 ( 31.7 and 45.5).  Paracetamol: 45.4 ( 35.4 and 56.5).  Gender: not reported.  Time after disease onset: stated no.  Stage: post acute.  Inclusion criteria: Post CHIK arthritis patients.  Exclusion criteria:  To reject informed consent to participate into trial.  Patients having rheumatoid arthritis, osteoarthritis, and other degenerative diseases. | Chloroquine: 150 mg (a white capsule).  Paracetamol: 500  mg (red capsule )  Dosage: daily single dose.  Follow up: 8 days.  Aim: antiviral-inflammatory. | Pain relief assessed with visual analogue scale. | Identifier trial number: not stated.  Sample size estimation a priori: No.  Conduction trial date: August 2012  Sponsor: not stated  Role of sponsor: not applied.  Conflict of interest: not declared. |
| Ravindran 2011 ^(43)^  Country: India | Parallel design  Two arms | Time after disease onset: more than 1 year after the onset of CHIK fever.  Stage: chronic.  72 adult patients with persistent CHIK arthritis (persistent polyarthritis for > 1 year after the onset of CHIK fever either in 2008 or 2009 fulfilling both epidemiological [typical narration, records] and serological (anti-  CHIKV IgM/IgG antibody by ELISA)) were screened | Methotrexate, sulfasalazine and hydroxychloroquine or monotherapy hydroxychloroquine.  Both groups received oral prednisolone (tapered off and discontinued  at 6 weeks). Rescue medications were monitored.  Aim: antiviral-inflammatory. | DAS28 (primary efficacy), HAQ (Indian version) and pain VAS (0–100 mm) [secondary efficacies] were assessed every 4 weeks for 24 weeks. | Identifier trial number: not stated.  Sample size estimation a priori: not stated.  Conduction trial date:  Sponsor: not stated.  Role of sponsor: not applied  .  Conflict of interest: not stated.  Data gathered from Conference’s meeting. |
| Padmakumar 2009 ^(42)^ Country: India. | Parallel design  Four arms | Sample size: 120  Age (mean (Standard deviation):  Group A: 50.07 (10.49) Group B: 49.63 (11.10) Group C: 50.83 (11.38) Group D: 47.10 (9.24)  Gender (Female):  Group A: 77%  Group B: 80%  Group C: 80%  Group D: 80%  Time after disease onset: within 6 weeks after onset fever.  Stage: acute  Adult patients (20-80 years) with classical features of sudden onset of fever (within 6 weeks) and joint pains, with inflammatory polyarthritis affecting both major and/or minor joints of upper and/or lower limbs, and IgM qualitative ELISA for CHIK who were enrolled. Normal routine laboratory was an inclusion criteria (hemoglobin, creatinine, white blood count, amino alanine transferase). Exclusion criteria: Pregnancy, significant renal or hepatic disease; class III/IV angina pectoris,  Uncontrolled hypertension, diabetes mellitus or congestive  cardiac failure, stroke or transient ischemic attack; allergy to paracetamol, pregnancy and lactation, rheumatoid arthritis, gout, gastric or duodenal ulcers, gall stones, influenza vaccination within prior 12 months, treatment with antiarrhythmics or cytotoxic medications and hepatic insufficiency. | **Comparison groups**:  Group A: aceclofenac alone; Group B: aceclofenac plus hydroxychloroquine; Group C: aceclofenac plus prednisolone  Group D: all three agents.  **Dosage**:  1. Aceclofenac (200 mg/day).  2. Hydroxychloroquine  (400 mg/day). 3. Prednisolone (10 mg/day).  Duration of treatment: 6 weeks  **Follow up**: 12 weeks. Weekly follow-ups followed by a 6 weeks drug-free follow-up with visits at week 8 and week 12.  Cointerventions:  All patients were given pantoprazole once daily for 12 weeks.  Paracetamol at dosage of 500 mg up to thrice by day.  Aim: antiviral-inflammatory. | Efficacy variables:  Visual analog scale for pain.  20- point modified Barthel index for activities of daily living  Instrumental activities of daily living . | Identifier trial number: not stated.  Sample size estimation a priori: No.  Conduction trial date: from November 2007 to June 2008.  Sponsor: Ministry of Health and Family Welfare, Government of Kerala and  National Rural Health Mission, Kerala.  Conflict of interest: authors declared no conflict. |

MSK-P: persistent musculoskeletal pain following acute CHIKV infection

CHIK: acute CHIKV infection.

VAS: visual analog scale
