# Supplementary material for: Interventions for treating patients with chikungunya virus infection-related rheumatic and musculoskeletal disorders: A systematic review
Source: PLoS One. 2017 Jun 13;12(6):e0179028. doi: 10.1371/journal.pone.0179028 (PMC5469465; doi:10.1371/journal.pone.0179028)
Supplement: S2 Table — (DOCX) [file pone.0179028.s004.docx]

**Table 2. Risk of bias assessment**

| **Reference** | **Random sequence generation (selection**  **bias)** | **Allocation concealment (selection bias)** | **Blinding of participants and personnel**  **(performance bias)**  **All outcomes** | **Blinding of outcome assessment (detection**  **bias)**  **All outcomes** | **Incomplete outcome data (attrition bias)**  **All outcomes** | **Selective reporting (reporting bias)** | **Other bias** | **Note** |
| --- | --- | --- | --- | --- | --- | --- | --- | --- |
| Chopra 2014 ^(44)^ | Unclear | Unclear | Unclear | Unclear | Low | Low | High |  |
| De Lamballerie 2008 ^(41)^ | Unclear | Unclear | Unclear | Unclear | Unclear | Unclear | Unclear |  |
| Ahmed 2012 ^(45)^ | Low | Unclear | High | High | Low | High | Bias in the presentation of the data. | After formation of the groups the white capsule group was given  chloroquine and the red capsule group was given paracetamol |
| Ravindran 2011 ^(43)^ | Unclear | Unclear | High | High | Unclear | Unclear | Low | Only the abstract was available. Drug doses not reported. |
| Padmakumar 2009 ^(42)^ | Unclear | Unclear | High | High | Low | High | High |  |
