# Supplementary material for: Interventions for treating patients with chikungunya virus infection-related rheumatic and musculoskeletal disorders: A systematic review
Source: PLoS One. 2017 Jun 13;12(6):e0179028. doi: 10.1371/journal.pone.0179028 (PMC5469465; doi:10.1371/journal.pone.0179028)
Supplement: S3 Table — (DOCX) [file pone.0179028.s005.docx]

SoF Tables. GRADE evidence profile

**Table 1. Summary of findings of meloxicam vs chloroquine**

| **Question: Should meloxicam vs chloroquine be used for rheumatic disorders in patients with an infection caused by the chikungunya virus? Bibliography:** Chopra A, Saluja M, Venugopalan A. Effectiveness of chloroquine and inflammatory cytokine response in patients with early persistent musculoskeletal pain and arthritis following chikungunya virus infection.  Arthritis & Rheumatology 2014;66(2):319-26. | | | | | | | | | | | |
| --- | --- | --- | --- | --- | --- | --- | --- | --- | --- | --- | --- |
| **Quality assessment** | | | | | | | **Summary of Findings** | | | | |
| **Participants (studies) Follow up** | **Risk of bias** | **Inconsistency** | **Indirectness** | **Imprecision** | **Publication bias** | **Overall quality of evidence** | **Study event rates (%)** | | **Relative effect** (95% CI) | **Anticipated absolute effects** | |
|  |  |  |  |  |  |  | **With chloroquine** | **With Meloxicam** |  | **Risk with chloroquine** | **Risk difference with Meloxicam** (95% CI) |
| **Pain relief** (CRITICAL OUTCOME; measured with: visual analog scale; range of scores: 0-10; Better indicated by lower values); range of scores: 0-10; Better indicated by lower values) | | | | | | | | | | | |
| 70 (1 study^1^) 24 weeks^6^ | serious^2^ | no serious inconsistency | no serious indirectness | very serious^3^ | undetected | ⊕⊝⊝⊝ **VERY LOW**^2,3^ due to risk of bias, imprecision | 38^5^ | 32^4^ | **-** | The mean pain relief in the control groups was **-4.2 points** | The mean pain relief in the intervention groups was **0.24 higher** (0.81 lower to 1.29 higher) |
| **Global health status or health related quality of life** (CRITICAL OUTCOME; measured with: Health Assessment Questionnaire; range of scores: 0-24; Better indicated by lower values); range of scores: 0-24; Better indicated by lower values) | | | | | | | | | | | |
| 70 (1 study^1^) 24 weeks^6^ | serious^2^ | no serious inconsistency | no serious indirectness | very serious^3^ | undetected | ⊕⊝⊝⊝ **VERY LOW**^2,3^ due to risk of bias, imprecision | 38 | 32 | **-** | The mean global health status or health related quality of life in the control groups was **-3.1 points** | The mean global health status or health related quality of life in the intervention groups was **0.31 lower** (2.06 lower to 1.44 higher) |
| **Adverse event** (CRITICAL OUTCOME) | | | | | | | | | | | |
| 70 (1 study^1^) 24 weeks^6^ | serious^3^ | no serious inconsistency | no serious indirectness | very serious^7^ | undetected | ⊕⊝⊝⊝ **VERY LOW**^3,7^ due to risk of bias, imprecision | 7/38  (18.4%)^8^ | 5/32  (15.6%) | **RR 0.85**  (0.30 to 2.42) | **184 per 1000**^8^ | **28 fewer per 1000** (from 129 fewer to 262 more) |

^1^ People with early persistent musculoskeletal pain and arthritis following chikungunya virus infection. Bavi (district of Sholapur in south central India) during the epidemic.
^2^ Downgraded one level due to limitations in the trial design or execution (unclear selection and performance bias).
^3^ Downgraded two levels due to imprecision (very low sample with an impact in the precision of the effect estimates).
^4^ Meloxicam: (7.5-mg tablet/once daily after lunch.
^5^ Chloroquine: 250-mg tablet/once daily after lunch.
^6^ At study completion.
^7^ Downgraded two levels due to imprecision (low sample and number of events with an impact in the precision of the effect estimates).
^8^ Assumed risk is based on the risks for the control group.

**Table 2. Summary of findings of chloroquine vs paracetamol**

| **Question: Should chloroquine vs paracetamol be used for treating rheumatic disorders in patients with an infection caused by the chikungunya virus? Bibliography:** Ahmed M, Shantharam N, Reddy YJV. Randomized clinical trial in Chikungunta arthritis cases. Journal of Evolution of Medical and Dental Ssciences 2012;1(5):841-7. | | | | | | | | | | | |
| --- | --- | --- | --- | --- | --- | --- | --- | --- | --- | --- | --- |
| **Quality assessment** | | | | | | | **Summary of Findings** | | | | |
| **Participants (studies) Follow up** | **Risk of bias** | **Inconsistency** | **Indirectness** | **Imprecision** | **Publication bias** | **Overall quality of evidence** | **Study event rates (%)** | | **Relative effect** (95% CI) | **Anticipated absolute effects** | |
|  |  |  |  |  |  |  | **With Paracetamol** | **With Chloroquine** |  | **Risk with Paracetamol** | **Risk difference with Chloroquine** (95% CI) |
| **Pain relief** (CRITICAL OUTCOME; assessed with: Visual analogue scale ^1^) | | | | | | | | | | | |
| 86 (1 study^2^) 8 days | serious^3^ | no serious inconsistency | no serious indirectness | very serious^4^ | undetected | ⊕⊝⊝⊝ **VERY LOW**^3,4^ due to risk of bias, imprecision | 27/43  (62.8%)^6,7^ | 41/43  (95.3%)^5^ | **RR 1.52**  (1.2 to 1.93) | **628 per 1000**^6,7^ | **327 more per 1000** (from 126 more to 584 more) |
| **Global health status or health related quality of life** - not reported | | | | | | | | | | | |
| - | - | - | - | - | - | See comment | - | - | **-** | See comment | See comment |
| **Adverse event** - not reported | | | | | | | | | | | |
| - | - | - | - | - | - | See comment | - | - | **-** | See comment | See comment |

^1^ Trial authors reported this outcome as binary variable.
^2^ Participants having post chikungunya arthritis in an urban health centre of PESIMSR, Kuppam (India).
^3^ Downgraded one level due to limitations in the trial design or execution (unclear selection and performance bias).
^4^ Downgraded two levels due to imprecision (low sample and number of events with an impact in the precision of the effect estimates).
^5^ Chloroquine: 155 mg//single dose.
^6^ Paracetamol: 500 mg/single dose.
^7^ Assumed risk is based on the risks for the control group.

**Table 3. Summary of findings of chloroquine vs placebo**

| **Question: Should chloroquine vs placebo be used for treating rheumatic disorders in patients with an infection caused by the chikungunya virus? Bibliography:** De Lamballerie X, Boisson V, Reynier JC, Enault S, Charrel RN, Flahault A, et al. On chikungunya acute infection and chloroquine treatment. Vector borne and zoonotic diseases (Larchmont, N.Y.) 2008;8(6):837-9. | | | | | | | | | | | |
| --- | --- | --- | --- | --- | --- | --- | --- | --- | --- | --- | --- |
| **Quality assessment** | | | | | | | **Summary of Findings** | | | | |
| **Participants (studies) Follow up** | **Risk of bias** | **Inconsistency** | **Indirectness** | **Imprecision** | **Publication bias** | **Overall quality of evidence** | **Study event rates (%)** | | **Relative effect** (95% CI) | **Anticipated absolute effects** | |
|  |  |  |  |  |  |  | **With Placebo** | **With Chloroquine** |  | **Risk with Placebo** | **Risk difference with Chloroquine** (95% CI) |
| **Pain relief (Acute period)** (CRITICAL OUTCOME; measured with: not reported; Better indicated by lower values) | | | | | | | | | | | |
| 54 (1 study^1^) 25 days^6^ | serious^2^ | no serious inconsistency | no serious indirectness | very serious^3^ | undetected | ⊕⊝⊝⊝ **VERY LOW**^2,3^ due to risk of bias, imprecision | 27^5^ | 27^4^ | **-** | The mean pain relief (acute period) in the control groups was **points**^7^ | The mean pain relief (acute period) in the intervention groups was **1.46 higher** (0.00 lower to 2.92 higher) |
| **Pain relief (Chronic period)** (CRITICAL OUTCOME; assessed with: Telephone interview (yes or not)) | | | | | | | | | | | |
| 54 (1 study^1^) 200 days^10^ | serious^2^ | no serious inconsistency | no serious indirectness | very serious^8^ | undetected | ⊕⊝⊝⊝ **VERY LOW**^2,8^ due to risk of bias, imprecision | 6/27  (22.2%)^5^ | 16/27  (59.3%)^4^ | **RR 2.67**  (1.23 to 5.77)^9^ | **222 per 1000**^5^ | **371 more per 1000** (from 51 more to 1000 more) |
| **Global health status or health related quality of life** - not reported | | | | | | | | | | | |
| - | - | - | - | - | - | See comment | - | - | **-** | See comment | This trial mentioned no this outcome. |
| **Adverse events** (CRITICAL OUTCOME) | | | | | | | | | | | |
| 54 (1 study^1^) 25 days | serious^2^ | no serious inconsistency | no serious indirectness | very serious^3^ | undetected | ⊕⊝⊝⊝ **VERY LOW**^2,3^ due to risk of bias, imprecision | 0/27  (0%)^5,9^ | 7/27  (25.9%)^4^ | **RR 15.00**  (0.90 to 250.24) | ^5,9^ | **-** |

^1^ Location: Reunion Island. Participants acute febrile arthralgia, and diagnosed within less than 48 hours.
^2^ Downgraded one level due to limitations in the trial design or execution (unclear selection and performance bias).
^3^ Downgraded two levels due to imprecision (very low sample with an impact in the precision of the effect estimates).
^4^ Chloroquine: 600 mg (one dose) at day 1, 600 mg (300 mg twice daily) at days 2 and 3, and 300 mg at days 4 and 5 (total dose: 2,400 mg; duration of treatment: 5 days).
^5^ Placebo
^6^ First period
^7^ Estimate effect calculated using P= 0.05. It was based on reported by trial authors "“The mean duration of febrile arthralgia was 4.3 days with no statistical difference between the chloroquine and placebo groups (4.7 and 3.9 days, respectively).
^8^ Downgraded two levels due to imprecision (low sample and number of events with an impact in the precision of the effect estimates).
^9^ Assumed risk is based on the risks for the control group
^10^ Second period

**Table 4. Summary of findings of DMARDs vs Hydroxychloroquine**

| **Question: Should Disease-Modifying Anti-Rheumatic (DMARD) therapy vs hydroxychloroquine monotherapy be used for treating rheumatic disorders in patients with an infection caused by the chikungunya virus? Bibliography:** Ravindran V, Alias G. Efficacy of combination DMARD therapy vs. hydroxychloroquine monotherapy in persistent chikungunya arthritis: a 24-week randomised controlled study.  Indian Journal of Rheumatology 2011;6(3 SUPPL. 1):S5. | | | | | | | | | | | |
| --- | --- | --- | --- | --- | --- | --- | --- | --- | --- | --- | --- |
| **Quality assessment** | | | | | | | **Summary of Findings** | | | | |
| **Participants (studies) Follow up** | **Risk of bias** | **Inconsistency** | **Indirectness** | **Imprecision** | **Publication bias** | **Overall quality of evidence** | **Study event rates (%)** | | **Relative effect** (95% CI) | **Anticipated absolute effects** | |
|  |  |  |  |  |  |  | **With Hydroxychloroquine monotherapy** | **With Disease-Modifying Anti-Rheumatic (DMARD) therapy** |  | **Risk with Hydroxychloroquine monotherapy** | **Risk difference with Disease-Modifying Anti-Rheumatic (DMARD) therapy** (95% CI) |
| **Pain relief** (CRITICAL OUTCOME; measured with: visual analog scale; range of scores: 0-100; Better indicated by lower values); range of scores: 0-100; Better indicated by lower values) | | | | | | | | | | | |
| 72 (1 study^1^) 24 weeks^6^ | serious^2^ | no serious inconsistency | no serious indirectness | very serious^3^ | undetected | ⊕⊝⊝⊝ **VERY LOW**^2,3^ due to risk of bias, imprecision | 35^5^ | 37^4^ | **-** | The mean pain relief in the control groups was **60.8 points** | The mean pain relief in the intervention groups was **14.80 lower** (19.12 to 10.48 lower) |
| **Global health status or health related quality of life** (CRITICAL OUTCOME; measured with: HAQ (Indian vesion); Better indicated by lower values) | | | | | | | | | | | |
| 72 (1 study^1^) 24 weeks^6^ | serious^2^ | no serious inconsistency | no serious indirectness | very serious^3^ | undetected | ⊕⊝⊝⊝ **VERY LOW**^2,3^ due to risk of bias, imprecision | 35^5^ | 37^4^ | **-** | The mean global health status or health related quality of life in the control groups was **1.88 points** | The mean global health status or health related quality of life in the intervention groups was **0.74 lower** (0.92 to 0.56 lower) |
| **Adverse events** (CRITICAL OUTCOME) | | | | | | | | | | | |
| 72 (1 study^1^) 24 weeks^6^ | serious^2^ | no serious inconsistency | no serious indirectness | very serious^7^ | undetected | ⊕⊝⊝⊝ **VERY LOW**^2,7^ due to risk of bias, imprecision | 0/35  (0%)^5,8^ | 1/37  (2.7%)^4^ | **RR 2.84**  (0.12 to 67.53) |  | **-** |

^1^ Participants with chronic persistent chikungunya arthritis.
^2^ Downgraded one level due to limitations in the trial design or execution (unclear selection and performance bias).
^3^ Downgraded two levels due to imprecision (very low sample with an impact in the precision of the effect estimates).
^4^ DMARD (at fixed dose of methotrexate 15 mg/week, sulfasalazine 1 g daily, and HCQ 400 mg daily) plus oral prednisolone (initial dose; 7.5 mg daily, tapered off and discontinued at 6 weeks).
^5^ Hidroxychloroquine: dose optimized to 400 mg/day plus oral prednisolone (initial dose; 7.5 mg daily, tapered off and discontinued at 6 weeks).
^6^ Calicut, India
^7^ Downgraded two levels due to imprecision (low sample and number of events with an impact in the precision of the effect estimates).
^8^ Assumed risk is based on the risks for the control group.

**Table 5. Summary of findings of aceclofenac monotherapy vs aceclofenac plus other pharmacological interventions**

| **Question: Should aceclofenac monotherapy vs aceclofenac plus other pharmacological interventions be used for rheumatic disorders in patients with an infection caused by the chikungunya virus?^1^ Bibliography:** Padmakumar B, Jayan JB , Menon RMR, Krishnankutty B, Payippallil R, Nisha RS. Comparative evaluation of four therapeutic regimes in chikungunya arthritis: A prospective randomized parallel-group study. Indian Journal of Rheumatology, 2009,4(3),94. | | | | | | | | | | | |
| --- | --- | --- | --- | --- | --- | --- | --- | --- | --- | --- | --- |
| **Quality assessment** | | | | | | | **Summary of Findings** | | | | |
| **Participants (studies) Follow up** | **Risk of bias** | **Inconsistency** | **Indirectness** | **Imprecision** | **Publication bias** | **Overall quality of evidence** | **Study event rates (%)** | | **Relative effect** (95% CI) | **Anticipated absolute effects** | |
|  |  |  |  |  |  |  | **With Aceclofenac plus other pharmacological interventions** | **With Aceclofenac monotherapy** |  | **Risk with Aceclofenac plus other pharmacological interventions** | **Risk difference with Aceclofenac monotherapy** (95% CI) |
| **Pain relief** (CRITICAL OUTCOME) | | | | | | | | | | | |
| 0 (1 study) 12 weeks^5^ | serious^2^ | no serious inconsistency | no serious indirectness | very serious^3^ | undetected | ⊕⊝⊝⊝ **VERY LOW**^2,3^ due to risk of bias, imprecision | - | 0^4^ | **-** | - | See footnote^6^ |
| **Global health status or health related quality of life** (CRITICAL OUTCOME) | | | | | | | | | | | |
| 0 (1 study) 12 weeks^5^ | serious^2^ | no serious inconsistency | no serious indirectness | very serious^3^ | undetected | ⊕⊝⊝⊝ **VERY LOW**^2,3^ due to risk of bias, imprecision | - | 0^4^ | **-** | - | See footnote^6^ |
| **Adverse events** - not mentioned | | | | | | | | | | | |
| - | - | - | - | - | - | - | - | - | **-** | - | This outcome was not mentioned. |

^1^ Either aceclofenac plus hydroxychloroquine or aceclofenac plus prednisolone or aceclofenac plus prednisolone and hydroxychloroquine.
^2^ Downgraded one level due to limitations in the trial design or execution (unclear selection and performance bias).
^3^ Downgraded two levels due to imprecision (very low sample with an potential impact in the precision of the effect estimates).
^4^ 30 participants
^5^ 6-weeks treatment period and 6-weeks drug-free.
^6^ This issue was reported narratively. Trial authors reported only P values.
